# Urologic Manifestations of Nonrelaxing Pelvic Floor Dysfunction: Insights on Clinical Workup and Management

**DOI:** 10.1007/s11934-025-01290-4

**Published:** 2025-10-16

**Authors:** Andrew S. Afyouni, Narmina Khanmammadova, Alireza Bozorgi, Akhil K. Das, Joel Gelman, Zhina Sadeghi

**Affiliations:** Irvine Department of Urology, University of California, Los Angeles, California USA

**Keywords:** Non-relaxing pelvic floor dysfunction, Voiding dysfunction, Dyssynergic voiding, Pelvic floor dysfunction, Lower urinary tract symptoms, Functional bladder outlet obstruction

## Abstract

**Purpose of Review:**

Non-relaxing pelvic floor dysfunction (NR-PFD) is a poorly understood and underdiagnosed cause of voiding dysfunction in patients without clear anatomic or neurologic obstruction. Symptoms may include pelvic pain, urinary complaints, defecatory dysfunction, and sexual issues, but their variability makes NR-PFD challenging to recognize and manage. This review focuses on the urologic manifestations of NR-PFD and outlines current diagnostic and treatment strategies.

**Recent Findings:**

Video urodynamics and surface EMG, alongside focused physical examination, are key tools for diagnosing NR-PFD. Pelvic floor physical therapy remains the first-line treatment, with strong evidence supporting its efficacy across sexes. Adjunctive options, including biofeedback, trigger point injections, botulinum toxin, and sacral neuromodulation, can benefit patients with refractory symptoms. Cognitive behavioral therapy and integrative modalities are also increasingly utilized.

**Summary:**

NR-PFD is an underrecognized cause of functional bladder outlet obstruction and complex LUTS. Management should be individualized and multidisciplinary. Future studies are needed to standardize diagnostic criteria and refine treatment algorithms.

## Introduction

Pelvic floor dysfunction (PFD) refers to a range of symptoms and anatomic abnormalities stemming from impaired pelvic floor muscle function. Presentations may include hypertonicity, hypotonicity, poor organ support (e.g., prolapse), muscle incoordination, or combinations thereof [1]. These muscles are essential for supporting pelvic and intra-abdominal organs, including the bladder, urethra, prostate, vagina, uterus, anus, and rectum, and play critical roles in continence and sexual function [2]. Proper relaxation and coordination are key to efficient voiding, defecation, and sexual activity.

Non-relaxing pelvic floor dysfunction (NR-PFD) is a subset of PFD marked by impaired muscle relaxation or paradoxical contraction, leading to pelvic pain, voiding and defecatory dysfunction, and sexual issues. Despite its prevalence, NR-PFD remains poorly understood and underdiagnosed. Literature has largely focused on its link to defecatory disorders [3] and chronic pelvic pain [2], often using overlapping terms such as pelvic floor tension myalgia, piriformis syndrome, levator ani spasticity, dyssynergic defecation, and pelvic floor dyssynergia.

In contrast, the urologic manifestations of NR-PFD have received relatively little attention in the literature and are frequently mismanaged in clinical practice due to lack of awareness. To this end, we conducted a comprehensive review of the scientific literature published between 2005 and 2025, with a focus on studies evaluating voiding dysfunction associated with NR-PFD. This review aims to highlight the urologic symptomatology, clinical implications, diagnostic challenges, and current treatment strategies for NR-PFD within a urologic framework.

## Role of the Pelvic Floor for Urinary Continence

At rest, tonic activity in the pelvic floor and external urethral sphincter maintains urethral closure pressure above bladder pressure to preserve continence. Effective voiding, by contrast, requires: (1) sustained detrusor contraction, (2) complete and sustained relaxation of the urethral sphincters, (3) coordinated pelvic floor muscle relaxation, and (4) absence of mechanical obstruction (e.g., urethral stricture, diverticulum, pelvic trauma).

The pelvic floor muscles, in particular, must fully relax and act in synchrony to permit descent of the bladder neck and opening of the urethra, allowing for efficient urine expulsion [4]. In individuals with NR-PFD, this coordinated relaxation fails to occur and the pelvic floor muscles remain contracted during voiding, effectively resulting in a functional bladder outlet obstruction (BOO).

Unlike detrusor-sphincter dyssynergia (DSD), which results from suprasacral spinal lesions, NR-PFD occurs in neurologically intact individuals and lacks structural or neurogenic abnormalities. It is also distinct from other non-neurogenic, non-structural causes of dyssynergic voiding, such as dysfunctional voiding (DV) and poor relaxation of the external sphincter (PRES). While DV typically involves both the external sphincter and pelvic floor involvement, NR-PFD selectively affects only the pelvic floor muscles [5, 6].

Importantly, NR-PFD does not require the presence of hypertonicity. Hypertonic pelvic floor dysfunction may be considered a subtype of NR-PFD; however, patients with NR-PFD can exhibit either normal or increased resting pelvic floor tone.

The hallmark feature of NR-PFD is the inability of these muscles to adequately relax during voiding. Due to limited studies specific to NR-PFD, this review also references data on hypertonic pelvic floor dysfunction, given the overlap in pathophysiology and treatment strategies.

## Pathophysiology

The most widely accepted theory implicates maladaptive learned behaviors, where individuals chronically contract their pelvic floor muscles to suppress urination or defecation [2, 7]. These patterns often originate during early childhood as part of toilet training, a period when the neural circuits governing pelvic floor control are highly plastic and susceptible to disruption. Instinctual behaviors such as excessive withholding or an inability to relax during voiding may persist into adulthood, often reinforced by occupational demands, avoidance of incontinence, or lifestyle factors [7]. Over time, this habitual suppression can result in persistent muscle activation and impaired voluntary relaxation.

Because anorectal and bladder control share similar neuromuscular mechanisms, several studies have drawn connections between functional defecatory disorders and the dyssynergic voiding seen in NR-PFD. In a case–control study of 28 women with functional defecatory symptoms, Klingele et al. found that 82% reported at least two urinary symptoms, and 57% reported four or more, including hesitancy, straining, interrupted stream, and incomplete emptying. Uroflowmetry revealed longer intervoid intervals and delayed time to peak urinary flow, consistent with NR-PFD [3].

Pelvic floor nonrelaxation and hypertonicity may also develop in response to chronic visceral pain syndromes. Conditions such as interstitial cystitis/bladder pain syndrome, irritable bowel syndrome, endometriosis, atrophic vaginitis, and vulvodynia have all been linked to pelvic floor overactivity via peripheral and central sensitization, nociceptive hypersensitivity, and referred myofascial pain [2]. Dyspareunia, in particular, may reinforce involuntary muscle contraction and promote a chronic state of non-relaxation, especially when intercourse is continued despite discomfort. In parallel, a history of childhood or adult sexual trauma has been associated with higher rates of chronic pelvic pain and pelvic floor dysfunction [8].

Trauma from obstetric events or pelvic surgery, such as mesh implantation, fascial fixation, or suturing to pelvic muscles, may also initiate pelvic floor dysfunction through local tissue injury and muscle spasm [7]. In one series, Gehrich et al. described postoperative urinary retention and pelvic floor hypertonicity following endometriosis excision, hypothesizing a compensatory spastic response that increased outlet resistance [9]. Similarly, Dietz et al. documented neuromuscular injury to the levator ani and pelvic support structures after vaginal delivery, contributing to post-partum dysfunction [10].

In many cases, no single causative event is identified. Rather, NR-PFD likely results from a constellation of behavioral, anatomic, neurologic, and psychosocial contributors. Once established, the condition may be further perpetuated by chronic pain–related factors, including sleep disturbance, anxiety, and depression, complicating both diagnosis and management.

## History and Symptomatology

When NR-PFD is suspected, a careful history and physical examination can help distinguish it from other disease processes. From a urologic perspective, the hallmark presenting symptom of NR-PFD is voiding dysfunction in the absence of any identifiable anatomic or neurologic obstruction. This dysfunction may include both storage and voiding symptoms. Storage symptoms typically consist of urinary frequency, urgency, and nocturia, while voiding symptoms may involve hesitancy, straining, weak or intermittent stream, a sensation of incomplete emptying, or urinary retention.

Like Klingele et al., Ackerman et al. identified a patient group with myofascial frequency syndrome (MFS), marked by LUTS without bladder pain or urgency incontinence typical of IC/BPS or OAB. These patients experienced urinary frequency, urgency without leakage, and bladder pressure similar to that of OAB-dry. Most exhibited non-relaxing pelvic floor hypertonicity on EMG and showed improvement with myofascial release-based pelvic floor physical therapy (PFPT) or biofeedback. Key symptoms included incomplete emptying, frequency, pelvic discomfort, and straining, without urgency, nocturia, incontinence, or bladder-specific pain [11, 12].

Other studies have reported that some patients with NR-PFD also experience dysuria or suprapubic discomfort, particularly when pelvic floor muscles fail to adequately relax during attempted voiding [13, 14]. These symptoms often resemble those of bladder outlet obstruction or overactive bladder, and in many cases, standard urologic evaluations fail to reveal a definitive cause, prompting further investigation into pelvic floor dysfunction as an underlying etiology.

Beyond the urinary tract, many individuals with NR-PFD report concomitant bowel symptoms, including constipation, obstructed defecation, excessive straining, and a persistent sensation of incomplete bowel evacuation [3, 15]. These symptoms may stem from the same underlying pelvic floor incoordination. In some patients, bowel dysfunction precedes urinary complaints and may date back to childhood, suggesting a shared or evolving pathophysiologic process.

NR-PFD is often linked to chronic, dull, and poorly localized pelvic pain that may radiate to the groin, back, or thighs. It is worsened by activities like sitting, walking, or intercourse and often described as a deep ache or pelvic floor tension myalgia [6]. Einig et al. noted higher rates of lower urinary tract symptoms in patients with pelvic myofascial pain and hypertonic pelvic floor [16]. However, pain is not universal; Ackerman et al. found some patients with voiding dysfunction and non-relaxing pelvic floor muscles did not report pain [11].

Sexual dysfunction is common, especially in women, presenting as dyspareunia, post-coital ache, or reduced sexual well-being [17]. Men may report perineal discomfort, ejaculatory pain, or pelvic heaviness [18]. These symptoms are likely due to chronic pelvic floor muscle contraction or myofascial trigger points.

Psychosocial history is crucial and should include sensitive inquiry about trauma or abuse, which is common in patients with complex pelvic floor disorders [2, 19]. In a study of 118 patients with dyssynergic defecation, 29% of men and 32% of women reported physical abuse, and 22% reported sexual abuse. Most experienced significant impacts on social life, work, sex, and family relationships, though the study focused primarily on defecatory symptoms [20].

## Physical Exam

A thorough, trauma-informed physical exam is essential in evaluating suspected NR-PFD, especially in the absence of standardized diagnostic criteria. The goal is to assess voluntary pelvic floor muscle control and identify signs of myofascial pain, hypertonicity, or incoordination, while ruling out structural or neurologic causes [1].

In women, the exam begins with visual inspection of the vulva, perineum, and anus, followed by assessment of voluntary contraction typically described as “squeezing to stop urine flow”, which should lift the perineum. Improper straining may lead to perineal descent and requires gentle correction or visual feedback [1]. Cotton swab testing helps localize vulvar allodynia, especially in vulvodynia [2, 21].

For men, inspection of the external genitalia and perineum is followed by assessment of the bulbocavernosus reflex and evaluation of perineal sensation. Patients may be asked to perform a pelvic floor contraction (“squeeze the anus or lift the scrotum”) while the examiner palpates for proper activation and relaxation. Tenderness, increased tone, or trigger points in the bulbospongiosus, ischiocavernosus, or levator ani muscles (palpated transrectally) may suggest myofascial involvement. Reproduction of pelvic, penile, scrotal, or rectal pain during these maneuvers is diagnostic for pelvic floor myalgia [1, 15, 22].

Speculum and bimanual exams assess for mucosal atrophy, prolapse, and pelvic masses in women, but digital palpation remains central in all patients. Vaginal or rectal exams evaluate tone, tenderness, trigger points, and contract-relax ability across pelvic floor muscles including the levator ani and obturator internus [22]. A rectal exam is critical for both sexes to assess sphincter tone, the coccyx, and rule out pain sources such as fissures or abscesses [1, 15].

Pelvic floor strength can be graded manually; lack of relaxation or involuntary tightening may indicate dysfunction [23, 24]. Palpation can trigger referred pain or urgency consistent with myofascial trigger points [6].

A focused neurologic exam should assess lower limb strength, reflexes, perineal sensation (S2–S4), and anal wink to exclude neuropathy [1]. Given the high prevalence of past trauma in these patients, exams should be conducted with clear communication and patient consent at each step to ensure a trauma-informed approach.

## Diagnostic Testing

While a thorough clinical examination may be sufficient to identify NR-PFD for some patients, further diagnostic testing is often warranted to rule out alternative or coexisting conditions. Depending on the symptom profile, workup should include evaluation for colorectal, gynecologic, neurologic, or inflammatory disorders that may contribute to or mimic NR-PFD [25]. In patients with complex presentations or inconclusive findings, objective assessment is essential, and referral to another specialist should be considered. Initial urologic evaluation should include basic laboratory studies such as a complete blood count (CBC), basic metabolic panel (BMP), and urinalysis with reflex urine culture. Additional diagnostic modalities may include urodynamic studies, uroflowmetry with post-void residual measurement, pelvic floor electromyography, cystoscopy, pelvic ultrasonography, and/or magnetic resonance imaging [26–28].

### Urodynamics

Urodynamic studies (UDS), especially multichannel pressure-flow studies with pelvic floor EMG, are key tools for evaluating NR-PFD. In complex or refractory cases, video UDS provide added value in diagnosing voiding dysfunction by integrating assessment of BOO, detrusor function, and pelvic floor behavior [5]. In a study of 207 women with BOO symptoms, Kuo et al. found pelvic floor obstruction in 51.2% of cases, defined by specific pressure-flow criteria and distal urethral narrowing on imaging [29]. Chuang et al. reported that bladder base elevation during filling was associated with higher voiding pressures and uncoordinated pelvic floor EMG activity [30].

Peng et al. also distinguished dysfunctional voiding (DV) from poor relaxation of external sphincter (PRES), finding that DV showed higher rates of BOO and detrusor overactivity, while PRES was associated with weaker contractility and less DO, highlighting NR-PFD as a distinct entity involving pelvic floor muscles rather than the urethral sphincter alone [5].

Surface EMG, the most commonly used method, involves placing adhesive electrodes around the anus and primarily measures levator ani activity, making it well-suited for assessing NR-PFD [31]. While less invasive than needle EMG, it has limitations such as signal interference from poor contact or moisture. Needle EMG, though more specific for urethral sphincter evaluation, can be uncomfortable and may trigger reflex spasms, limiting its utility [32].

When paired with VUDS, EMG helps localize obstruction and clarify the underlying dysfunction. Interpretation challenges remain, but innovations like high-density intravaginal EMG with 64-channel arrays offer more precise mapping of pelvic floor activity and may enhance future diagnosis of NR-PFD [33, 34].

### Uroflowmetry

Several studies have evaluated the diagnostic utility of uroflowmetry in identifying NR-PFD or pelvic floor muscle spasm, particularly in male patients with lower urinary tract symptoms. Abnormal flow patterns, such as staccato or interrupted streams, may suggest pelvic floor dysfunction, but their sensitivity and specificity are limited when uroflowmetry is used in isolation. Lasri et al. found uroflowmetry alone had 60.4% sensitivity and 75.1% specificity for detecting straining to void, with specificity improving to 84.9% when combined with surface EMG, though sensitivity remained similar [35]. Wenske et al. similarly showed that abnormal uroflow patterns correlated with EMG-confirmed pelvic floor dysfunction in only 33–50% of cases, underscoring the limitations of relying on uroflowmetry alone [36].

While uroflowmetry and PVR assessment are useful initial tests in the evaluation of voiding dysfunction. When clinical suspicion is high, these tests should be supplemented with pelvic floor EMG or urodynamic studies to improve diagnostic accuracy.

### Cystoscopy

While it does not directly diagnose pelvic floor dysfunction, cystoscopy enables direct visualization of the lower urinary tract and is useful for identifying secondary changes that may result from chronic dysfunctional voiding or increased outlet resistance, such as bladder wall trabeculation, diverticula, and, less commonly, bladder neck hypertrophy or urethral strictures. These changes can develop in response to persistently elevated voiding pressures associated with non-relaxing pelvic floor activity.

Cystoscopy also allows for the exclusion of coexisting or mimicking structural conditions, such as urethral stricture, bladder tumor, or prostatic enlargement, entities that cannot be reliably differentiated through uroflowmetry or urodynamic studies alone [32].

### Ultrasonography

Ultrasound can visualize pelvic floor movement, urethral displacement, and bladder neck mobility during voiding, offering insight into muscle coordination [37, 38]. 3D imaging improves accuracy by measuring bladder neck descent and levator hiatus area [39].

It moderately correlates with EMG and rectal exams, with good reliability. Bladder wall thickening (> 3 mm) may suggest chronic outlet resistance in NR-PFD [40]. Though useful, ultrasound lacks pressure or EMG data, limiting its role in fully characterizing obstruction.

### Magnetic Resonance Imaging

Magnetic resonance imaging (MRI) offers high-resolution, multiplanar visualization of pelvic floor anatomy and is primarily used as an adjunctive tool in the evaluation of NR-PFD. MRI can accurately delineate pelvic floor muscle morphology, allowing for the assessment of muscle atrophy, hypertrophy, or avulsion, and can clarify the spatial relationships of pelvic organs—features that may be particularly useful in complex, postsurgical, or anatomically ambiguous cases.

Dynamic MRI, including MR defecography, enables visualization of pelvic floor motion during straining or voiding. However, its correlation with functional symptoms or urodynamic findings remains limited. Moreover, the lack of standardized protocols and diagnostic criteria for NR-PFD restricts its broader application in routine clinical assessment [41].

## Treatments

Managing urologic symptoms from NR-PFD is complex and must be tailored to each patient’s unique presentation. Due to the variability in symptoms, especially when pelvic pain and non-urologic issues overlap, a multidisciplinary, multimodal approach is essential. (Table 1) Collaboration among urologists, other specialists (colorectal, gynecology, etc.), pelvic floor physical therapists, pain specialists, and behavioral health providers ensures comprehensive care. Key goals include restoring normal voiding, reducing symptoms, and preventing complications such as UTIs, bladder dysfunction, hydronephrosis, and long-term renal damage.

**Table 1:** Summary of literature evaluating available treatment modalities

Article	Study Design & Treatment Modality	Patients	Symptom Distribution	Findings/Response Rate
[59] **Aboseif S., et al., 2002** *Sacral neuromodulation as an effective treatment for refractory pelvic floor dysfunction*	Prospective; sacral neuromodulation	54 women 10 men	Various forms of voiding dysfunction for whom other forms of therapy had failed: • ~ 69% with frequency, urgency, urge incontinence (Group 1) • ~ 31% with idiopathic, nonobstructive, chronic urinary retention requiring intermittent catheterization (Group 2) • ~ 64% also had chronic pelvic pain associated with other voiding symptoms (Group 3)	Mean follow-up time was 24 (6–36) months; Overall, 80% reported ≥ 50% improvement in symptoms and quality of life: • Group 1: 77% reported symptoms and quality of life (QOL) improvement, willing to recommend this therapy to a friend/relative • Group 2: 90% were able to void spontaneously and reported ≥ 50% improvement in QOL, agreeing that they would recommend this procedure to a friend/relative • Group 3: Non-significant improvement in mean pain score from preoperative 5.8 to postoperative 3.7
[46]** Cornel E.B., et al., 2005** *The effect of biofeedback physical therapy in men with chronic pelvic pain syndrome type III*	Prospective; pelvic floor biofeedback re-education program	33 men	Pelvic pain or discomfort for at least 3 months, the National Institutes of Health Chronic Prostatitis Symptom Index (NIH-CPSI) > 15	Outcomes evaluation after 6 to 8 treatment sessions: • ~ 6% (n = 2/33) drop-out • Decrease in CPSI score post-treatment in 97% (n = 32/33) of patients • Improvement in the subdomain of micturition in 81% (n = 21/33) of patients • Improvement in the subdomain of pain in 87% (n = 27/33) of patients • Improvement in the subdomain of quality of life in 74% (n = 23/33) of patients; with 29% (n = 13/33) improving 5 points or more • Significant decrease in the mean value of the pelvic muscle tonus from 4.9 μV preoperatively to 1.7 μV postoperatively on EMG • At the patient level, 94.4% (17/18) of patients experienced a decrease in pelvic muscle tonus from preoperative to postoperative measurements; it remained unchanged in 1 patient
[52]** Langford C.F., et al., 2007** *Levator ani trigger point injections: An underutilized treatment for chronic pelvic pain*	Prospective; trigger point injections	18 females	Females with minimum 6 months of chronic pelvic pain and specific palpable trigger points of levator ani + pelvic floor muscle exercises post-injection; all had tried prior medical therapy including narcotic pain medication and amitryptyline, 7/18 received instillation therapies for a pre-existing diagnosis of Interstitial Cystitis (IC)	Mean follow-up time was 3 (3–12) months & success was defined as ≥ 50% decrease in pain as measured on Visual Analog Scale (VAS), ≥ 60% improvement in individual patient global satisfaction (PGS) and cure rates (PGC) visual scores: • Improvement in 72% (n = 13/18) of patients following the first injection • 33% (n = 6/18) reported being completely pain-free • Overall decrease in VAS from 88.8% to 36%, and mean PGS and PGC scores, both of 63% (P < 0.0001) • In 7 women with IC, 71% had a ≥ 50% decrease in their VAS, a PGS of 58% and PGC of 62%
[62]** Lee S.W.H., et al., 2008** *Acupuncture versus Sham Acupuncture for Chronic Prostatitis/Chronic Pelvic Pain*	Prospective randomized; acupuncture vs. sham acupuncture (at non-acupoints)	44 patients randomized to acupuncture group vs. 45 to sham group	Patients meeting US NIH consensus criteria for Chronic Prostatitis (CP)/Chronic Pelvic Pain Syndrome (CPPS), with a total score ≥ 15 on the NIH-CPSI and experiencing symptoms during ≥ 3 of the previous 6 months	Primary response characterized as a 6-point drop in the NIH-CPSI total score (range: 0–43) between the initial assessment and week 10: • Response in 73% (n = 32/44) of patients in acupuncture group compared to 47% (n = 21/45) in sham group (p = 0.02) • Improvement in score for 4.5 points more on average in acupuncture group after 10-week treatment (p = 0.03) • No significant difference in the average reduction of NIH-CPSI total score between responders to acupuncture and to the sham (15 vs. 10 points, respectively) • No significant difference in complete symptom resolution after 10-week treatment in acupuncture vs. sham participants (40.9% vs. 22.2%, p = 0.07) with both groups experiencing reduction in NIH-CPSI total scores from baseline (p < 0.001) • significant difference in the proportion of participants achieving ≥ 50% improvement in Global Response Assessment scores between the acupuncture and sham groups (65.9% vs. 40%, p = 0.02) • Long-term response (24 weeks post-procedure) in 32% (n = 14/44) of acupuncture group participants vs. in 13% (n = 6/45) of sham group (p = 0.04)
[44]** Minardi D., et al., 2010** *The Role of Uroflowmetry Biofeedback and Biofeedback Training of the Pelvic Floor Muscles in the Treatment of Recurrent Urinary Tract Infections in Women With Dysfunctional Voiding: A Randomized Controlled Prospective Study*	Prospective randomized; uroflowmetry biofeedback (group 1; n = 24) vs. biofeedback training of the pelvic floor muscles (group 2; n = 21) vs. uroflowmetry biofeedback combined to biofeedback training of the pelvic floor muscles (group 3, n = 20) vs. no treatment (group 4, n = 21)	86 women	• Female patients with dysfunctional voiding diagnosed using multichannel video urodynamic investigation and perineal ultrasound • ≥ 3-year history of recurrent UTIs • ≥ 3 symptomatic UTI episodes within a 12-month period • UTI diagnosis based on clean-catch urine specimens sent for urinalysis and culture • Nulliparous women • Age < 40 years • No genital prolapse • No prior surgery for incontinence or pelvic procedures • No behavioral causes of recurrent UTIs (e.g., low water intake, delayed voiding, bowel issues, sexual activity)	• Significant decrease in the incidence of storage and emptying symptoms at 3-, 6- and 12-months post-treatment in group 1, 2, and 3 (p < 0.05) with response remaining consistent during the study period; no decrease in these symptoms in group 4 • Significant increase in mean flow rate, flow time, and voided volume in all three treatment groups (p < 0.05), with improvements maintained up to 12 months • Significant decrease in postvoid residual urine in treated groups (p < 0.05), sustained at 12-months • Significant reduction in mean opening detrusor pressure and detrusor pressure at maximum flow in treated groups (p < 0.05), stable during follow-up; no significant change in untreated group • Significant decrease in mean and maximum urethral closure pressure in treated groups (p < 0.05); no change in untreated group • Group 3 showed a significantly greater decrease in urethral pressures compared to groups 1 and 2 (p < 0.05) • Prevalence of UTI significantly decreased in all treated groups post-treatment and remained stable during follow-up (p < 0.05); no change in untreated group • At 24 months: 55% of patients had storage/emptying symptoms and voiding patterns similar to baseline; UTI incidence returned to baseline in 45% of patients across groups 1–3
[60]** Jadav A.M., et al., 2013** *Does sacral nerve stimulation improve global pelvic function in women?*	Prospective; sacral neuromodulation	43 women	Evaluation of the effect of sacral neuromodulation on non-bowel related (i.e., co-existing pelvic floor dysfunction) symptomatology in patients undergoing sacral neuromodulation for fecal incontinence; At baseline: • 100% (n = 43/43) reported urinary symptoms • 81.4% (n = 35/43) reported vaginal symptoms • 85.7% (n = 36/43) reported some sexual dysfunction	• Significant improvement in fecal incontinence, irritable bowel syndrome-related symptoms, bowel-related quality of life, and bowel-related sexual health (all p < 0.01) • Significant improvement in symptoms of vaginal prolapse (p = 0.05), vaginal pain, and sensation (p < 0.05) • Improvement in overactive bladder symptoms (p = 0.005) and urinary-related quality of life (p < 0.05) • 58.1% reported overall health improvement, mainly in bowel evacuation and vaginal comfort • Among sexually active women, 53.3% reported improved overall sex life, with significant improvements in both vaginal and bowel-related sexual health (p < 0.005)
[61] **Kucuk E.V., et al., 2015** *Effectiveness of acupuncture on chronic prostatitis-chronic pelvic pain syndrome category IIIB patients: A prospective, randomized, nonblinded, clinical trial*	Prospective, randomized; the medical treatment group (group 1, n = 28) and the acupuncture treatment group (group 2, n = 26)	54 men	Minimum 12 weeks of pelvic pain underwent clinical and microbiological tests and diagnosed as an NIH category IIIB CPPS	Mean follow-up was 28 weeks from the baseline (range, 20–43 weeks): • Significant decrease in pain scores in both groups after treatment (p < 0.01), with a greater decrease in the acupuncture group vs. the medical treatment group (6.65 vs. 3.89; p < 0.05) • Greater improvement in the NIH-CPSI total score in acupuncture group (12.54 vs. 6.43; p < 0.01) • 89.3% (n = 25/28) of patients were classified as treatment responders in the acupuncture group • Greater decreases observed in group 2 vs. group 1 in urinary symptom score and quality of life scores, though not statistically significant (p > 0.05)
[55]** Zoorob D., et al., 2015** *A pilot randomized trial of levator injections versus physical therapy for treatment of pelvic floor myalgia and sexual pain*	Prospective, randomized; pelvic floor physical therapy (PT) or levator-directed trigger-point injections (LTPI)	29 women: 17 had PT, 12 had LTPI; to be included minimum one LTPI or three PT sessions required	Female patients with pelvic floor myalgia (PFM), sex-related pain	Pain assessed at 1 month posttreatment; with Levator-based pain assessed using a numeric rating scale (NRS) and the Patient Global Impression of Improvement (PGI-I) scale and sexual function assessed using the Female Sexual Function Index (FSFI): • Pain (NRS): Both groups had similar pain reduction (mean change: PT 4.47, LTPI 4.67; p = 0.8); ≥ 50% improvement in 59% (PT) vs. 58% (LTPI) • Shorter time to pain relief with LTPI (4.4 vs. 7.3 weeks; p = 0.01) • Sexual Function (FSFI): Greater improvement with PT (+ 8.87 vs. + 4.00; p = 0.04), particularly in sexual pain domain (p = 0.02) • Post-treatment sexual dysfunction resolution in 35% of PT and 8% of LTPI patients • Similar global Improvement between groups (PGI-I): PT 2.50, LTPI 2.17; p = 0.5
[56]** Morrissey D., et al., 2015** *Botulinum Toxin A Injections into Pelvic Floor Muscles under Electromyographic Guidance for Women with Refractory High-Tone Pelvic Floor Dysfunction: A 6-Month Prospective Pilot Study*	Prospective; electromyography-guided onabotulinumtoxinA injections	21 women	Chronic pelvic pain and high-tone pelvic floor dysfunction who have failed conventional therapy: • 42.9% had interstitial cystitis/bladder pain syndrome • 66.7% had vulvodynia	• Improvement in the Global Response Assessment scale was observed in 61.9% of patients at 4 weeks, and 80.9% at 8-, 12-, and 24-weeks post-injection compared to baseline • 58.8%—83.3% of sexually active participants reported less dyspareunia over time (4 to 24 weeks) • Significant decrease in dyspareunia VAS score from 7.8 at baseline to 5.6 at 12 weeks (p = 0.011) and 5.4 at 24 weeks (p = 0.004) • Significant improvement in Female Sexual Distress Scale scores assessing sexual dysfunction at 8, 12, and 24 weeks (p < 0.05 for all) • Improved quality of life assessed via Short-Form 12 Health Survey (SF-12): better physical composite scores at all time points (p < 0.05); improvement in mental composite scores at 12 and 24 weeks (p = 0.012) • Significant decreases in resting and maximum contraction pelvic floor pressures at all visits on vaginal manometry (p < 0.05) • Decrease in PFM tenderness on digital assessment at all follow-ups vs. baseline (p < 0.001)
[64]** Goldfinger C., et al., 2016** *Effectiveness of Cognitive-Behavioral Therapy and Physical Therapy for Provoked Vestibulodynia: A Randomized Pilot Study*	Prospective, randomized; CBT (n = 10) vs. comprehensive PT (n = 10)	20 women	Women with provoked vestibulodynia	• ≥ 30% clinically meaningful reduction in pain during intercourse reported by ~ 70% of CBT group and 80% of PT group post-treatment (p = 0.64) • Significant decreases in pain during the cotton swab test from pre- to post-treatment in PT group; by 6-months, both groups experienced significant decreases • Significant decreases in pain during gynecologic exam compared to baseline, no difference between two modalities • Improved ability to maintain intercourse without interruption due to pain • Improvements in sexual functioning observed only among CBT completers; sexual satisfaction improved from baseline in both groups • Both groups showed significant reductions in pain catastrophizing and increased perceived control over pain compared to baseline; no between group differences • Reduced percentages of daily activities resulting in pain, and painful intercourse attempts • Improvements largely sustained at 6-month follow-up
[58]** Dessie S.G., et al., 2019** *A randomized, double-blind, placebo-controlled trial of onabotulinumtoxin A trigger point injections for myofascial pelvic pain*	Prospective, randomized, placebo-controlled, double-blind; trigger point injections of 200 units of onabotulinumtoxin A (n = 30) or 20 mL saline (n = 29)	59 women	Women with myofascial pelvic pain reporting pain ≥ 6 on a 10-point VAS ≥ 50% of the time and had pain on palpation ≥ 6 on the VAS in ≥ 1 of 6 pelvic floor muscle groups	• No significant difference in pain on palpation of the most painful pelvic floor muscle between intervention and placebo groups at 2, 4, or 12 weeks • No significant difference in overall pelvic pain reduction (VAS) between groups at 4 and 12 weeks (p = 0.16 for both) • A higher proportion of the intervention group reported symptom improvement on the Patient Global Impression of Improvement index at 4 weeks (p = 0.03); the rates were comparable at 12 weeks (p = 0.10) • Significant improvement in Pelvic Floor Distress Inventory score in the placebo group compared to intervention at 2 weeks (p = 0.01); no significant difference at 4 or 12 weeks (p = 0.19 and p = 0.11, respectively)
[53]** Hui J., et al., 2020** *A novel, nonopiod-based treatment approach to men with urologic chronic pelvic pain syndrome using ultrasound-guided nerve hydrodissection and pelvic floor musculature trigger point injections*	Retrospective analysis; Weekly US-guided pelvic floor TPIs to the iliococcygeus, pubococcygeus, and puborectalis with 1% lidocaine while continuing PFPT. These patients also received peripheral nerve hydrodissection performed on the pudendal and posterior femoral cutaneous nerve. First two injections: combination of 1% lidocaine + dexamethasone. Final four injections: 1% lidocaine + traumeel (a homeopathic, plant-derived anti-inflammatory medication)	8 men	Men with urologic chronic pelvic pain syndrome (UCPPS) having already completed a trial of NSAIDs in combination with antibiotic therapy in addition to PFPT pre- and post-treatment	• Significant decrease in mean VAS scores from 3.3 (± 1.7) at pretreatment to 1.8 (± 1.4) posttreatment at 6-week follow-up (p < 0.05; 95% CI, 0.73–2.27) • Significant decrease in mean Functional Pelvic Pain Scale scores from 11.0 (± 8.0) at pretreatment to 6.3 (± 5.3) posttreatment 6-week follow-up (p < 0.05; 95% CI, 0.03–9.22)
[47]** Chiang C.H., et al., 2021** *Therapeutic efficacy of biofeedback pelvic floor muscle exercise in women with dysfunctional voiding*	Retrospective; examining changes in global response assessment (GRA), clinical symptoms, QOL index, and uroflowmetry parameters after 3 months of biofeedback PFPT	31 women	Female patients with dysfunctional voiding who underwent biofeedback PFPT	• 25 (81%) patients had successful outcomes (GRA ≥ 2) after 3 months of biofeedback PFPT • IPSS-T, IPSS-S, IPSS-V, PPBC, and QoL index significantly improved • Qmax, proportion of low Qmax, cQmax, VV, TBC, Qave, T-voiding, and T-Qmax significantly improved • Pre-PFPT: 7% of patients revealed a non-obstructive (bell-shaped) flow curve vs. 93% in obstructive pattern (Staccato-shaped: 58.1%; Plateau-shaped: 35.5%) o Post-PFPT 3 months: 29% patients were less obstructed (29%, Staccato-shaped: 19%; Plateau-shaped: 10%)
[50] **Volpe L. J., et al., 2023** *Objective Changes in Pelvic Floor Muscle Strength and Length in Women with High-Tone Pelvic Floor Dysfunction after Pelvic Floor Physical Therapy (RELAX Trial)*	Prospective cohort; measuring intravaginal closure force, levator hiatal dimension, and symptom severity across 6 sessions of PFPT	22 women	Women undergoing 6 sessions of PFPT for the diagnosis of hypertonic PFD	• No significant differences in mean vaginal closure force (3.27 ± 2.34 vs 3.67 ± 2.02 N, p = 0.18) • Significant increase in mean levator hiatal area was observed between visit 1 (13.71 ± 1.77 cm2) and visit 6 (14.43 ± 2.17 cm2, p = 0.05) • Significant increase in transverse diameter (3.83 ± 0.03 vs 3.95 ± 0.03 cm, p = 0.04) • Significant improvements in survey responses pertaining to genitourinary symptoms, pain, lower gastrointestinal symptoms and QOL measures after 6 sessions of PFPT
[54]** Lewis G.K., et al., 2023** *Trigger point injections followed by immediate myofascial release in the treatment of pelvic floor tension myalgia*	Retrospective; Pelvic floor trigger point injection (PFTPI; n = 22, 25%) vs. PFTPI with subsequent PFPT (n = 65, 75%)	87 women	Women with pelvic floor trigger points who failed initial PFPT and oral/transvaginal muscle relaxants	• Median pre-treatment VAS score was 8 for both groups • Post-treatment: o PFPTI only: 6 o PFPTI + PFPT: 4 o This was a median change in VAS score of 2 and 4, respectively (p = 0.042) • 77% patients who underwent PFTPI + PFPT had VAS score improvement ≥ 3 vs. 45% of patients who underwent PFTPI alone (p = 0.008)
[57] **Sprujit M.A., et al., 2024** *The Efficacy of Botulinum Toxin A Injection in Pelvic Floor Muscles in Chronic Pelvic Pain Patients: A Double-Blinded Randomised Controlled Trial*	Randomized, double‐blinded clinical trial; Botulinum Toxin A (BTA) injections vs. placebo injections, combined with PFPT	94 women	Women with chronic pelvic pain and increased pelvic floor muscle tone despite previous PFPT	• By 8 weeks, 15 participants (33%) had a ≥ 33% reduction in average pain score after BTA treatment vs. 9 participants (20%) after placebo treatment (odd ratio placebo/BTA 1.88; 95% CI 0.72–4.90, p = 0.19) • By 26 weeks, no significant difference was observed between groups (p = 0.05) • Pelvic floor resting activity decreased significantly after BTA treatment compared to placebo even past 26 weeks (p = 0.001) o This decrease did not correspond to a reduction in pain scores

### Pelvic Floor Physical Therapy, Biofeedback, and Manual Therapies

Pelvic floor physical therapy (PFPT) is the first-line treatment for NR-PFD and serves as the foundation of conservative management for urologic symptoms related to pelvic floor hypertonicity and/or impaired relaxation [42]. It is most effective when delivered by therapists trained in pelvic floor rehabilitation and incorporates interventions such as neuromuscular re-education, diaphragmatic breathing, core stabilization, and reverse Kegel exercises that enhance pelvic floor coordination and reduce muscle overactivity.

Biofeedback can enhance PFPT by helping patients identify and relax overactive pelvic muscles, especially when symptoms are thought to stem from learned behaviors [43]. In a randomized trial, Minardi et al. found that uroflowmetry-based biofeedback with pelvic muscle training significantly reduced UTIs and improved voiding parameters [44]. For patients with chronic pain, biofeedback may facilitate deeper muscle relaxation. Education on posture, behavioral triggers, and pelvic awareness is also essential [6, 22].

PFPT has demonstrated efficacy across sexes. For men, PFPT has shown to be an effective modality in improving high-tone pelvic floor dysfunction. Polackwich et al. observed that 79% of men achieved clinically meaningful improvement in NIH Chronic Prostatitis Symptom Index (NIH-CPSI) scores, including voiding-related metrics such as urinary frequency, incomplete emptying, sexual dysfunction, and pelvic pain with PFPT [45]. Additionally, biofeedback-assisted PFPT has demonstrated efficacy in reducing pelvic floor tone and symptom burden among men with both chronic pelvic pain and bothersome urinary symptoms [46].

In women, PFPT, when used alone or in combination with biofeedback and relaxation training, has demonstrated similarly high success rates in treating non-relaxing pelvic floor tonicity and lower urinary tract symptoms. In a prospective study, 81% of women achieved clinical and uroflowmetric improvements after 3 months of biofeedback-assisted PFPT [47]. Meta-analyses confirm PFPT as the most effective conservative treatment for urinary incontinence and broader LUTS in women with high-tone pelvic floors, improving symptom severity and urodynamic measures [48, 49]. The RELAX trial (Volpe et al.) demonstrated significant levator hiatal lengthening after six sessions of PFPT, accompanied by improvements in pelvic pain, bowel, bladder, and quality-of-life metrics [16, 50].

Experts in Torosis et al. unanimously endorsed PFPT as first-line therapy for hypertonic pelvic floor dysfunction, recommending 1–2 sessions weekly for 8–12 weeks. Following symptom improvement, patients should be advised to follow a home exercise regimen and undergo follow-up at 4–6 months [42].

### Medical/Pharmacological Therapies

Pharmacologic management is considered adjunctive and should be tailored to the patient’s most bothersome symptoms, as most medications do not directly address the underlying neuromuscular dysfunction and are not first-line treatments.

Muscle relaxants, such as oral baclofen, tizanidine, cyclobenzaprine, and diazepam, may reduce pelvic floor spasm and provide symptom relief. Diazepam suppositories are the most commonly prescribed vaginal formulation and are preferred over oral routes due to lower systemic absorption and longer local activity, resulting in fewer side effects [42].

Neuropathic pain agents, including amitriptyline, nortriptyline, gabapentin, and pregabalin, are often used for chronic pelvic pain, though tricyclic antidepressants may exacerbate bowel or bladder symptoms and are generally avoided in NR-PFD [2].

In patients with overlapping overactive bladder symptoms, anticholinergics (e.g., fesoterodine, tolterodine) and β3 agonists (e.g., mirabegron) may modestly improve urgency. However, these agents do not address pelvic floor dysfunction and are ineffective for voiding symptoms related to non-relaxation; their use should be limited to cases where urgency predominates.

### Trigger Point Injections

Trigger point injections (TPIs) are considered second-line or adjunctive therapies for NR-PFD, particularly in patients unresponsive to PFPT. A myofascial trigger point is a tender nodule within a taut muscle band, often seen in hypertonic muscles, though not all tight muscles exhibit localized tenderness [22, 51]. As such, TPIs are most appropriate when focal tender points can be reliably identified.

The standard technique involves palpating pelvic floor muscles perpendicular to their fibers to detect taut bands, then along the fibers to find the most symptomatic point. Local anesthetic (typically 0.25–0.5% bupivacaine) is injected into this site. Corticosteroids may be added but offer no proven advantage. Expert consensus favors an anesthetic alone [42].

TPIs do not directly relax muscle tone but may reduce pain and interrupt the guarding reflex, facilitating downstream pelvic floor relaxation. In one study, levator ani TPIs improved symptoms by > 50% in 72% of patients with chronic pelvic pain [52]. In men with urologic chronic pelvic pain syndrome and pelvic floor hypertonicity, ultrasound-guided TPIs to muscles such as the iliococcygeus, pubococcygeus, and hip adductors improved NIH-CPSI and pain scores after a short treatment course [53]. Similarly, in women, TPIs with local anesthetic reduced pelvic myalgia and urinary symptoms (e.g., urgency and frequency) in those refractory to physical therapy [54, 55].

Experts in Torosis et al. reached no consensus on the use of anesthesia during TPIs. Additionally, pudendal nerve blocks are not indicated for NR-PFD unless pudendal neuralgia is suspected or diagnostic clarification is needed; they are ineffective for pure muscle hypertonicity without neuropathic features [42].

### Botulinum Toxin A

Botulinum toxin A (BTXA) is considered a third-line option for refractory pelvic floor hypertonicity or myofascial pain unresponsive to PFPT or other conservative therapies. Per expert consensus, BTXA should be injected directly into hypertonic or non-relaxing muscles and used alongside continued PFPT. Injections are typically bilateral, and lack of response after 1–2 sessions should prompt transition to alternative treatments [42].

BTXA may reduce pain via both muscle relaxation and modulation of nociceptive [56]. However, randomized controlled trials in women with pelvic floor hypertonicity and chronic pelvic pain—groups overlapping with NR-PFD—have not shown BTXA to outperform placebo in improving pain, urinary symptoms, or global function, even when combined with PFPT [57, 58]. While minor improvements in resting pressure and quality of life have been reported, they have not translated into significant clinical benefit for urologic outcomes.

There is wide variability in dosing protocols. Reported regimens range from 10 to 50 units per muscle group, depending on the number and location of the targeted muscles. Total BTXA dosing should not exceed 360 units within a three-month period [42]. BTXA use in hypertonic or NR-PFD remains off-label, and further studies are needed to define its role in urologic symptom management.

### Sacral Neuromodulation

Sacral neuromodulation (SNM) may be considered for patients with persistent voiding dysfunction or pelvic pain unresponsive to conservative and pharmacologic therapies. In a cohort of 64 patients with refractory pelvic floor dysfunction, Aboseif et al. reported that 80% experienced > 50% improvement in symptoms and quality of life after SNM [6, 59]. Anecdotally, SNM has also been associated with improvements in bowel, vaginal, neuropathic, and sexual symptoms [60].

Despite these benefits, SNM is an invasive treatment and is not FDA-approved specifically for NR-PFD. In Torosis et al., 60% of experts supported its use only in patients with coexisting urgency, frequency, or retention who have failed less invasive measures. SNM should not be used as primary therapy for isolated NR-PFD, and patients must be counseled on its off-label use and expected outcomes [42].

### Integrative/Holistic Approaches: Acupuncture, At-Home Therapies, and Cognitive Behavioral Therapy

Complementary and integrative approaches may serve as valuable adjuncts, particularly in patients with persistent pelvic pain or stress-related symptom exacerbations. While data remain limited, some evidence supports the use of acupuncture in improving pain, urinary symptoms, and overall quality of life among patients with chronic pelvic floor dysfunction [61, 62].

First-line home-based therapies, including daily pelvic floor stretching, application of warm compresses, and relaxation-focused exercises such as yoga, are frequently recommended as first-line management options for patients [42]. These interventions are low-risk, accessible, and may reinforce pelvic floor relaxation and neuromuscular downregulation.

Cognitive behavioral therapy (CBT) has shown benefit in NR-PFD patients with chronic pelvic pain or psychosexual dysfunction. Its utility lies in targeting central sensitization, maladaptive coping, and psychosocial contributors [63, 64]. In Torosis et al., 80% of experts supported incorporating psychological counseling or CBT into the treatment algorithm, typically as a second-line option following PFPT [42].

## Conclusion

**Fig. 1:**
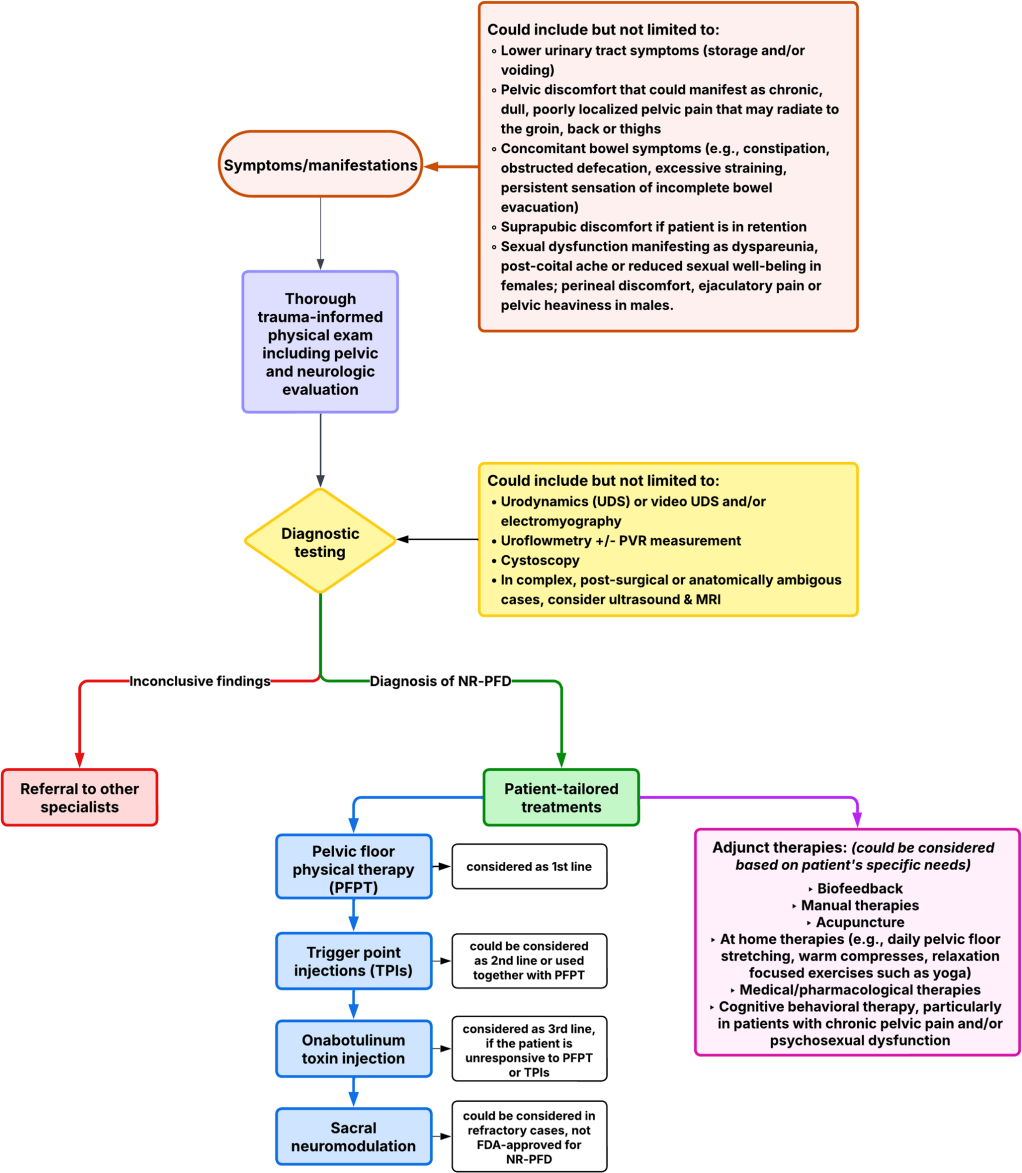
Algorithm for evaluation, diagnosis, and treatment of non-relaxing pelvic floor dysfunction (NR-PFD)

Non-relaxing pelvic floor dysfunction (NR-PFD) represents a complex, underrecognized cause of voiding dysfunction, particularly in patients without identifiable structural or neurologic abnormalities. Its heterogeneous symptom profile, often overlapping with other pelvic floor, gastrointestinal, and psychosocial conditions, complicates timely diagnosis and appropriate management. A high index of suspicion, combined with detailed clinical assessment and selective use of diagnostic modalities, is essential for identifying NR-PFD as an underlying etiology of lower urinary tract symptoms. Pelvic floor physical therapy remains the cornerstone of treatment, with a growing role for adjunctive interventions such as biofeedback, trigger point injections, neuromodulation, and integrative care approaches. (Fig. 1) Given the multifactorial nature of NR-PFD, effective management requires individualized, multidisciplinary care. Future research should prioritize the development of standardized diagnostic criteria, objective assessment tools, and evidence-based treatment algorithms to enhance patient outcomes.

## Key References

- Volpe, L. J. et al. Objective Changes in Pelvic Floor Muscle Strength and Length in Women with High-Tone Pelvic Floor Dysfunction after Pelvic Floor Physical Therapy (RELAX Trial). Urogynecology 29, 872–979 (2023).
  - Prospective study of patients with high-tone pelvic floor dysfunction undergoing six sessions of pelvic floor physical therapy, evaluating changes in levator muscle dimensions, vaginal closure force, and short-term effects on pain, genitourinary and lower gastrointestinal symptoms, and patient-reported quality of life.
- Lewis, G. K., Chen, A. H., Craver, E. C., Crook, J. E. & Carrubba, A. R. Trigger point injections followed by immediate myofascial release in the treatment of pelvic floor tension myalgia. Arch Gynecol Obstet 307, (2023).
  - Retrospective analysis of women with pelvic floor muscle tension assessing outcomes of pelvic floor physical therapy with myofascial release initiated immediately after pelvic floor trigger point injections, demonstrating greater pain improvement with this combined approach.
- Spruijt, M. A. et al. The Efficacy of Botulinum Toxin A Injection in Pelvic Floor Muscles in Chronic Pelvic Pain Patients: A Double-Blinded Randomised Controlled Trial. BJOG (2024) https://doi.org/10.1111/1471-0528.17991.17991.
  - A randomized double-blinded clinical trial evaluating the efficacy of Botox injection combined with pelvic floor muscle therapy in women with chronic pelvic pain, which did not support the use of Botox in this cohort.
- Torosis, M. et al. A Treatment Algorithm for High-Tone Pelvic Floor Dysfunction. Obstetrics and Gynecology 143, 595–602 (2024).
  - Evidence- and consensus-based guidelines developed by multidisciplinary experts, outlining a stepwise or concurrent multimodal treatment approach for high-tone pelvic floor dysfunction, emphasizing pelvic floor physical therapy as first-line therapy and identifying key treatment barriers.

## Data Availability

No datasets were generated or analysed during the current study.
